# Panic Attack Prediction Using Wearable Devices and Machine Learning: Development and Cohort Study

**DOI:** 10.2196/33063

**Published:** 2022-02-15

**Authors:** Chan-Hen Tsai, Pei-Chen Chen, Ding-Shan Liu, Ying-Ying Kuo, Tsung-Ting Hsieh, Dai-Lun Chiang, Feipei Lai, Chia-Tung Wu

**Affiliations:** 1 Graduate Institute of Biomedical Electronics and Bioinformatics National Taiwan University Taipei City Taiwan; 2 Department of Psychiatry En Chu Kong Hospital New Taipei City Taiwan; 3 Department of Computer Science and Information Engineering National Taiwan University Taipei City Taiwan; 4 Financial Technology Applications Program Ming Chuan University Taoyuan City Taiwan

**Keywords:** panic disorder, panic attack, prediction, wearable device, machine learning, lifestyle

## Abstract

**Background:**

A panic attack (PA) is an intense form of anxiety accompanied by multiple somatic presentations, leading to frequent emergency department visits and impairing the quality of life. A prediction model for PAs could help clinicians and patients monitor, control, and carry out early intervention for recurrent PAs, enabling more personalized treatment for panic disorder (PD).

**Objective:**

This study aims to provide a 7-day PA prediction model and determine the relationship between a future PA and various features, including physiological factors, anxiety and depressive factors, and the air quality index (AQI).

**Methods:**

We enrolled 59 participants with PD (*Diagnostic and Statistical Manual of Mental Disorders, 5th edition*, and the Mini International Neuropsychiatric Interview). Participants used smartwatches (Garmin Vívosmart 4) and mobile apps to collect their sleep, heart rate (HR), activity level, anxiety, and depression scores (Beck Depression Inventory [BDI], Beck Anxiety Inventory [BAI], State-Trait Anxiety Inventory state anxiety [STAI-S], State-Trait Anxiety Inventory trait anxiety [STAI-T], and Panic Disorder Severity Scale Self-Report) in their real life for a duration of 1 year. We also included AQIs from open data. To analyze these data, our team used 6 machine learning methods: random forests, decision trees, linear discriminant analysis, adaptive boosting, extreme gradient boosting, and regularized greedy forests.

**Results:**

For 7-day PA predictions, the random forest produced the best prediction rate. Overall, the accuracy of the test set was 67.4%-81.3% for different machine learning algorithms. The most critical variables in the model were questionnaire and physiological features, such as the BAI, BDI, STAI, MINI, average HR, resting HR, and deep sleep duration.

**Conclusions:**

It is possible to predict PAs using a combination of data from questionnaires and physiological and environmental data.

## Introduction

### Background

Panic disorder (PD) is a common mental disorder with a lifetime prevalence of about 1.6%-3.5% worldwide [1,2]. Its main characteristic is the fear of recurrent panic attacks (PAs) and loss of control, which leads to functional impairment. Patients suffering from PD often make frequent visits to the emergency department before formal diagnosis and psychoeducation. Functional impairment of PD can be avoidant behavior in terms of crowds, open spaces, traffic vehicles, or stressful situations. Severe PD cases [3] may become homebound. Accurate PA prediction may help clinicians to provide appropriate, timely treatment and to optimize personalized medicine.

A PA is typically an abrupt surge of intense fear reaching a peak within minutes, including 4 or more of the following symptoms: palpitations; sweating; trembling or shaking; sensations of shortness of breath or smothering; a feeling of choking; chest tightness; nausea or abdominal distress; dizziness or faintness; derealization (feelings of unreality) or depersonalization (being detached from oneself); fear of losing control, or going crazy; fear of dying; numbness or tingling sensation; chills; and heat sensational disturbance. A PA with fewer than 4 symptoms is called a limited panic attack (limited PA). Due to its high prevalence, the *Diagnostic and Statistical Manual of Mental Disorders, 5th edition* (DSM-5) [4], uses PA as a descriptive specifier across all mental disorders [5].

### Theory and Hypothesis

PAs are known to be triggered by psychological stress or specific occasions that induce a fear of being unable to escape (agoraphobia). However, so far, few studies have predicted recurrent PAs using real-life data. We hypothesize that recurrent PAs are related to multiple factors, including physiological, emotional, and personality factors. Cho et al [6] and Trushna et al [7] further observed a positive association between PAs and air pollution. To confirm these associations, we evaluated PAs from various domains to establish a more explainable model.

### Previous Work

Researchers have used a variety of data sources to predict PD severity and prognosis, including demographic features, clinical scales, diagnostic information, medical history, functional magnetic resonance imaging (fMRI), electrocardiogram (ECG), electroencephalogram (EEG), and genetic data, such as DNA methylation signatures [8]. In recent years, a few studies have begun to use watch-type computers, wearable devices, or physical challenge by CO_2_ [9] to predict PAs. However, there is no clear evidence showing which features are superior for prediction. It is also difficult to compare these studies due to the heterogeneity of study design, methods, and sample selections. Next, we give a brief review.

*Clinical questionnaires* with internal consistency and reliability are the tools widely used to predict PA and PD severity. These tools assess the participant's emotional and personality traits, for example, the Anxiety Sensitivity Index (ASI) [10,11], the State-Trait Personality Inventory (STPI) [12], Hamilton Depression Rating Scale (HAM-D), Beck Anxiety Inventory (BAI), and State-Trait Anxiety Inventory (STAI). Liu et al [13] used 11 predictors for PD recurrence from past demographic, clinical, and psychosocial factors, yielding a discriminative power C statistic of 72.8%. Most clinical questionnaires can be delivered at clinics or via internet-based approaches.

*fMRI* [14-17] compares areas of brain activation before and after a particular treatment, clarifies the structural change in PD, and predicts whether PD is comorbid with depression. However, predictions from different fMRI studies are inconsistent [18]. In addition, fMRI is expensive and complicates real-time PA prediction. It is, instead, an excellent tool by which to explore the psychopathology of PD.

An *EEG* detects specific patterns, such as slow waves in the θ-band, in PD patients, as shown in a study [19]. A review of EEG [20] summarized that PD tends to show decreased α-band power and increased β-band power, but the review did not yield an algorithm to predict PAs using EEG patterns.

*Wearable devices* are the most promising tool by which to detect PAs throughout the patient's daily life. Patients can wear smartwatches, rings, or headsets most of the time. Wearable devices using *ECG* data were used to evaluate PD in another 6 studies in a review [21]. Among these, results on statistical significance were inconsistent. However, some studies included Holter monitors as wearable devices, and they were not set in the patient's living environment, nor did they make use of the internet. In these studies, researchers found that heart rate variability (HRV) [22] can demonstrate the association between cardiac autonomic dysregulation and PD. Another survey by Cruz et al [23] used wearable and mobile systems to evaluate the severity of PA symptoms in correlation with physiological parameters. These parameters included the heart rate (HR), breathing rate, HRV, core temperature, and activities. However, it did not yield a model to predict PAs. The effect size and testing duration were both limited.

Jacobson et al [24] used a multilayered ensemble deep learning model paired with wearable *actigraph* units to passively sense data to predict deterioration in anxiety disorder symptoms. The result showed a balanced accuracy of 68.7% and an area under the curve of 69.6%. However, this study aimed to predict the long-term anxiety prognosis of PD rather than PAs. In addition, we could not correlate its time-sequence anxiety level with actigraphy. Sakamoto et al [25] used watches to detect PAs in 16 patients for 2 weeks. They found positive correlations between the PA frequency, locomotor activity (r=0.55), and Hamilton Anxiety Rating Scale (HAM-A) scores.

### Goal of This Study

The purpose of this study was to establish a real-time PA prediction model. Data sources included clinical scales, diagnostic information, wearable devices, and environmental factors. We also compared the prediction importance between different data sources.

## Methods

### Participants

We recruited 59 participants from the En Chu Kong Hospital, Taiwan, psychiatric clinics between June 2020 and April 2021. The inclusion criteria were (1) a primary diagnosis of PD by DSM-5, (2) age more than 20 years, and (3) a basic ability to navigate smartwatch and mobile phone apps. Civil law defines an age of 20 years as becoming an adult in Taiwan. Below this age, the study required additional ethical regulation and opinions from participants' legal guardians, making the process more complicated.

The exclusion criteria were (1) current substance abuse, (2) cardiopulmonary incapacity, (3) limited mental capacity or total mental incapacity, and (4) acute suicidal ideation. This study required sufficient mental capacity on the part of participants to cooperate by continuously wearing smartwatches, properly maintaining the smartwatches, and completing regular, valid online questionnaires. Limited mental capacity implies that the person has difficulty understanding, remembering, or using the information to make or communicate a decision. Our team evaluated the participants' mental capacity during the diagnostic interview (DI), Mini International Neuropsychiatric Interview (MINI), and the process of informed consent by certified psychiatrists and nurse practitioners. The information about acute suicidal ideation was obtained from DIs and responses to questions in MINI part A and the preassessment Beck Depression Inventory (BDI).

### Study Approval

This study was approved and monitored by the institutional review board (ECKIRB1090305) of En Chu Kong Hospital. The research team securely stored all data according to the agreement, and privacy was protected by the Graduate Institute of Biomedical Electronics and Bioinformatics at National Taiwan University, Taiwan.

### Data Collection

The data we collected contained physiological data, environmental data, and questionnaire data. We obtained physiological data from the wearable device, which captured the participants' steps, distance traveled, floors climbed, HR in different states, and time of different sleep stages. The HR states captured during the monitoring period included (1) the minimum HR values, (2) the maximum HR values, (3) the average HR during the past 7 days, and (4) the average HR at rest, all in beats per minute (bpm). The different stages of sleep captured included (1) deep, (2) light, (3) rapid eye movement (REM), and (4) awake stages, all in seconds.

We obtained environmental data from the Environmental Protection Administration's Environmental Open Data Platform. We located the nearest environmental monitoring station according to each participant's residential address. These data were the air quality index (AQI), SO_2_ subindex, CO subindex, particulate matter 1.0 microns (PM_1.0_) subindex, NO_2_ subindex, and particulate matter 2.5 microns (PM_2.5_) subindex. We collected these data every day to map the data from the smartwatches.

The questionnaire involved the Panic Disorder Severity Scale (PDSS), BDI, BAI, STAI, and MINI. Psychiatric professionals use MINI to screen the participants for mental comorbidities at the first DI.

#### PDSS-SR, Chinese Version

Houck et al [26] developed the PDSS Self-Report version (PDSS-SR) in 2002, with the Chinese version [27] validated in 2020. This assessment includes 7 items: PA frequency, distress, anticipatory anxiety, agoraphobic fear, avoidance of panic-related bodily sensations, work impairment, and social impairment. Based on their rating on a 5-point scale, 0 indicated “not at all” and 1-4 indicated “mild,” “moderate,” “severe,” and “extreme,” respectively [27]. The first question in the PDSS-SR is, ”How many panic and limited-symptom attacks did you have during the week?“

The prediction model ground truth (labeling) was ”True“ if the first question to the PDSS-SR was answered with 1, 2, 3, or 4 and ”False“ if the answer was 0. We sought to detect whether participants had experienced any PAs in the previous week. The PDSS-SR was collected at 2-week intervals for 1 continuous year via a mobile app or over the phone.

#### BDI and BAI

The BDI II [28] measures the severity of depression using 21 questions. Each question has 4 choices (0, 1, 2, and 3): a higher score represents a more depressing description. The cut-off points of the sum are *minimal* (0-13), *mild* (14-19), *moderate* (20-28), and *severe* (29-63) depressive symptoms. The BAI II [29] measures the severity of anxiety using 21 questions. Each question has 4 choices: 0, *not at all*; 1 *mildly, but it didn't bother me much*; 2, *moderately—it wasn't pleasant at times*; and 3, *severely—it bothered me a lot*. The cut-off points of the sum are *minimal* (0-7), *mild* (8-15), *moderate* (16-26), and *severe* (26-63) depressive symptoms.

#### STAI-S and STAI-T

The STAI Chinese version [30,31] measures anxiety levels. The STAI differentiates the temporary condition of state anxiety (STAI-S) and the more general and long-standing quality of trait anxiety (STAI-T). The essential attributes evaluated by the STAI-S scale are feelings of tension, nervousness, and worry [30]. The 4-point STAI-S scale is as follows: 1, *not at all*; 2, *somewhat*; 3, *moderately* *so*; and 4, *very much so*. The 4-point STAI-T scale is as follows: 1, *rarely*; 2, *sometimes*; 3, *often*; and 4, *almost constantly*. The cut-off point is 41 for the STAI-S and 43 for the STAI-T for clinically significant anxiety state/trait symptoms.

Participants self-reported their STAI-S and STAI-T initially and every 2 weeks via a mobile app.

### System Architecture

**Figure 1:**
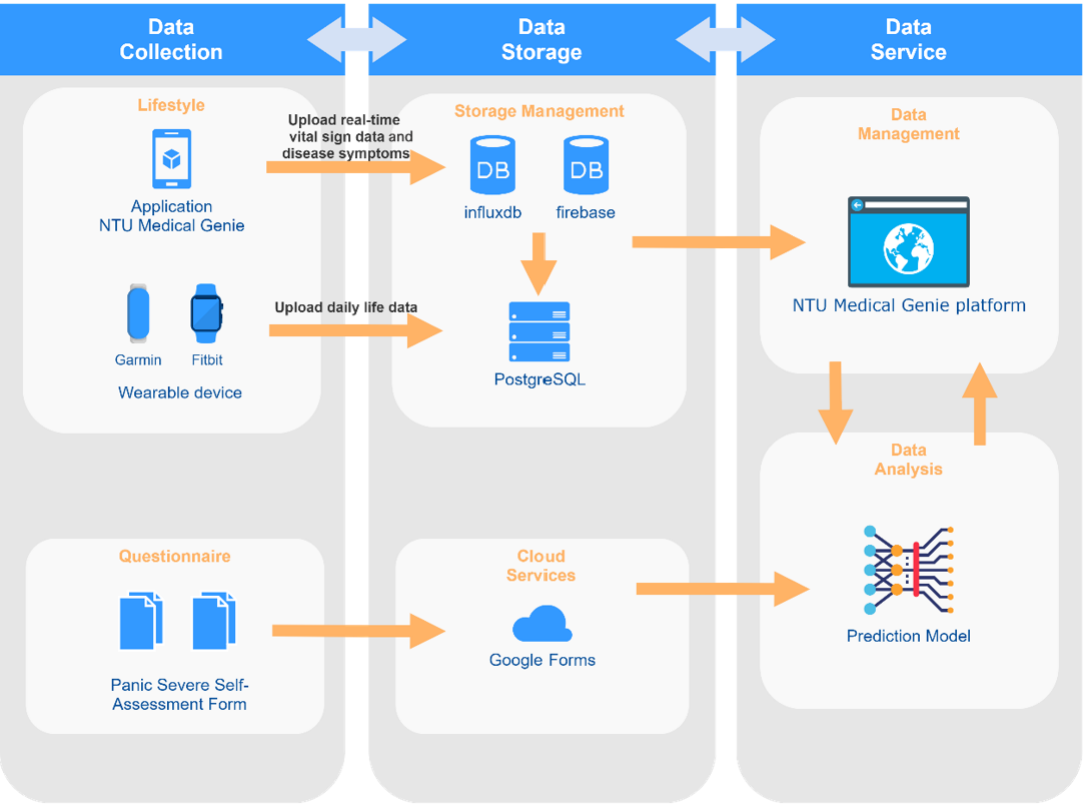
System architecture.

The PA prediction system architecture contained 3 parts: data collection, data storage, and data service, as shown in Figure 1. For data collection, we included lifestyle data (physiological data) and questionnaire data. The wearable device (Garmin Vívosmart 4) automatically collected the physiological information via Bluetooth and uploaded daily life data. In addition, we developed a smartphone app to collect real-time physiological data. Our team stored the daily life data in Postgres Structured Query Language and real-time physiological data in an influx database. Questionnaire data were collected via a Google form and stored in Google Drive.

We used the NTU Medical Genie platform for data service, management, and checking of participants' physiological data. Visualized data were also available on this platform, which helped the case manager to efficiently observe data. In addition, our team implemented the prediction model on the forum.

### Data Processing

**Figure 2:**
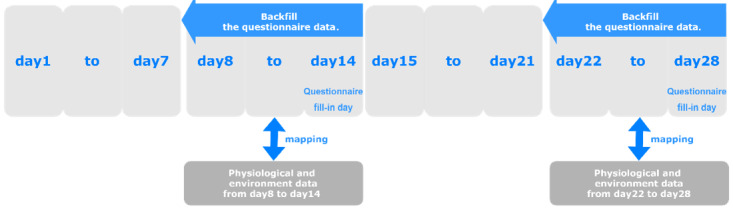
Data mapping process.

The data set we used to train the model was a combination of physiological data, environmental data, and questionnaire data. First, for missing values in the questionnaire data, we filled in the average value of each question for each participant. Second, Figure 2 illustrates how we mapped physiological and questionnaire data. Participants filled out the questionnaire every 2 weeks.

We used backward filling to go back 7 days based on the questionnaire-filling date, after which we combined the questionnaire data with the physiological and environmental data. Participants responded to questionnaires based on their status in the past 7 days. The label summarizes ”PA or not“; other situations reported in the questionnaire happened shortly before labeling time. In addition, 1 day corresponded to 1 data point, each of which serves as an individual observation for model training. If the label was true, we set each data point as true for 7 days and vice versa.

We experimented with several methods to mitigate the recall bias from questionnaires: First, the research assistants and clinicians followed up with the participants every 2 weeks over the telephone or through face-to-face interviews to ensure that the content of the questionnaires was consistent with the actual status in the previous week. Second, we examined the electronic medical records (EMRs) to determine whether the self-reported content (PA or not) was consistent with medical notes. The study duration was 1 year; thus, the backfilling method allowed participants to report their mood every 2 weeks rather than that in a more intense time frame—once a week or daily—to facilitate their acceptance and adherence to the study.

After mapping all the data, we removed all data points for which physiological or environmental data were missing. This resulted in 3249 data points from June 2020 to March 15, 2021, as the training set and 974 data points from March 16 to April 2021 as the test set. We set the training and test sets in different time frames because it is closer to the clinical scenario. We aim to deploy this model in the future to mixed samples, both familiar and new patients. With this arrangement, the test set performance would benefit from within-patient correlation and also patients unseen by the model.

In Textbox 1, we present the final set of features used in the model.

**Final set of features used in the study model.**

**Environmental factors**

- Air quality index (AQI)
- SO_2_ subindex
- CO subindex
- Particulate matter 1.0 microns (PM_1.0_) subindex
- NO_2_ subindex
- Particulate matter 2.5 microns (PM_2.5_) subindex

**Physiological factors**

- Steps
- Distance
- Floors
- Minimum heart rate (HR)
- Maximum HR
- Average HR
- Resting HR
- Sleep duration
- Deep sleep duration
- Light sleep duration
- Rapid-eye-movement (REM) sleep duration
- Awake duration

**Clinical questionnaires**

- Beck Depression Inventory (BDI)
- Beck Anxiety Inventory (BAI)
- State-Trait Anxiety Inventory (STAI); 40 answers
- Panic Disorder Severity Scale (PDSS); 1 answer as the ground truth
- Mini International Neuropsychiatric Interview (MINI)

### Classification Models

To predict PAs, we experimented with machine learning classifiers, including random forests, decision trees, linear discriminant analysis (LDA), adaptive boosting (AdaBoost), extreme gradient boosting (XGBoost), and regularized greedy forests (RGFs). We implemented these models using Python 3.6.10 libraries and Scikit-learn 0.23.1. We used 10-fold cross-validation and grid search for optimization of modeling. In the random forest example, we initially used a grid search to set up different combinations of hyperparameters. We tried “n_estimators: [50, 100, 200, 300], min_samples_split: [1, 2, 5, 10], min_samples_leaf: [1, 2, 5, 10],” with a total of 64 possible combinations of hyperparameters. After 10-fold cross-validation, we randomly split 10 parts of an equal amount of data in the training set. Later, we used 9 parts as training and 1 as validation in a rotation. Eventually, we averaged the F1 score of these 10 validation results and chose the best hyperparameter combination. The results of this process are shown in Table 1.

**Table 1:** Model hyperparameters.

Model	Hyperparameter	Value, n
Random forest	n_estimators	100
	min_samples_split	2
	min_samples_leaf	1
Decision tree	min_samples_split	2
	min_samples_leaf	1
LDA^a^	solver	lsqr
	shrinkage	auto
AdaBoost^b^	n_estimators	50
	learning_rate	1
XGBoost^c^	objective	binary:logistic
	learning_rate	0.0001
RGF^d^	max_leaf	1000
	algorithm	RGF_Sib
	test_interval	100

^a^LDA: linear discriminant analysis.

^b^AdaBoost: adaptive boosting.

^c^XGBoost: extreme gradient boosting.

^d^RGF: regularized greedy forest.

### Validation and Model Assessment

We used 20% of the training data to evaluate the model in terms of accuracy, sensitivity, specificity, and the F1 score. We also used the testing data set to assess the model's predictive ability with respect to data never seen by the training model.

We tried several percentages, and the split of 20% gave the highest accuracy of the training result. According to previous experience from machine learning, a 10%-30% range is ideal for optimization of modeling.

## Results

### Clinical Characteristics of Participants

Table 2 summarizes participant demographic factors and comorbidities according to MINI and the initial clinical questionnaires. Participant ages ranged from 20 to 74 years. The female-male ratio was 1.56. Nearly half (30/59, 51%) of the participants were comorbid with at least 1 psychiatric illness: agoraphobia (13/59, 22%) and general anxiety disorder (GAD; 19/59, 32%) were the 2 most common comorbidities. In addition, 4 (7%) of the 59 participants were comorbid with depression, and 4 (7%) were comorbid with posttraumatic stress disorder (PTSD). The initial mean range of the PDSS-SR was 8.2 (SD 5.3), indicating clinically significant PA symptoms. The initial mean BAI was 20.5 (SD 12.4), and the mean BDI was 13.6 (SD 9.8), revealing a state of mild-to-moderate anxiety and minimal-to-mild depression. The initial mean STAI-S score was 45.2 (SD 7.2), and the initial mean STAI-T score was 47.6 (SD 7.1). Both state and situational anxiety were clinically significant at the time of recruitment.

**Table 2:** Clinical characteristics of participants (N=59).

Characteristics			Value		Interpretation
**Age (years)**					
	Mean (SD)	46.2 (14.7)		Participant ages ranged from 20 to 74 years.	
	Range	20.1-74.8			
**Gender, n (%)**					
	Male	23 (39.0)		The female-to-male ratio was 1.56.	
	Female	36 (61.0)			
**Comorbidity, n (%)**					
	Agoraphobia	13 (22.0)		Nearly half (n=30, 51%) of the participants were comorbid with at least 1 psychiatric illness. Agoraphobia (n=13, 22%) and GAD (n=19, 32%) were the 2 most common comorbidities.	
	GAD^a^	19 (32.2)			
	Social anxiety disorder (SAD)	1 (1.7)			
	Major depressive disorder (MDD)	4 (6.8)			
	Bipolar disorder	1 (1.7)			
	PTSD^b^	4 (6.8)			
	Obsessive-compulsive disorder (OCD)	2 (3.4)			
	Others^c^	2 (3.4)			
	No comorbidity	29 (49.2)			
**Initial PDSS-SR^d^**					
	Mean (SD)	8.2 (5.3)		Clinically significant panic symptoms.	
	Range	0-23			
**Initial BDI^e^**					
	Mean (SD)	13.6 (9.8)		Minimal-to-mild depression.	
	Range	0–46			
**Initial BAI^f^**					
	Mean (SD)	20.5 (12.4)		Mild-to-moderate anxiety.	
	Range	1-44			
**Initial STAI-S^g^**					
	Mean (SD)	45.2 (7.2)		Clinically significant situational anxiety.	
	Range	33-69			
**Initial STAI-T^h^**					
	Mean (SD)	47.6 (7.1)		Clinically significant trait anxiety.	
	Range	32-65			

^a^GAD: general anxiety disorder.

^b^PTSD: posttraumatic stress disorder.

^c^Others: history of heroin use disorder (n=1, 1.7%), psychotic disorder (n=1, 1.7%).

^d^PDSS-SR: Panic Disorder Severity Scale Self-Report (>4 shows significant PD symptoms).

^e^BDI: Beck Anxiety Inventory (minimal, 0-13; mild, 14-19; moderate, 20-28; severe, 29-63).

^f^BAI: Beck Anxiety Inventory (minimal, 0-7; mild, 8-15; moderate, 16-25; severe, 26-63).

^g^STAI-S: State-Trait Anxiety Inventory state anxiety (scoring 20-80, >41 shows situational anxiety).

^h^STAI-T: State-Trait Anxiety Inventory trait anxiety (scoring 20–80, >44 shows trait anxiety).

### PDSS-SR Result

Of all 3249 data points in the training set, 2109 (64.91%) showed no PA (PDSS-SR Q1=0), 832 (25.61%) showed only mild-intensity PAs (PDSS-SR Q1=1), 231 (7.11%) showed moderate PAs (PDSS-SR Q1=2), 58 (1.79%) showed severe PAs (PDSS-SR Q1=3), and 52 (1.6%) showed extreme PAs (PDSS-SR Q1=4). In addition, 32 (68%) of 47 participants experienced at least 1 PA or limited symptoms, and 15 (32%) of 47 participants denied any PA or had limited symptoms. Of all 974 data points in the test set, 641 (65.8%) showed no PA, 267 (27.4%) showed mild PAs, 65 (6.7%) showed moderate PAs, 1 (0.1%) showed severe PAs, and none showed extreme PAs. In addition, 28 (54%) of 52 participants experienced at least 1 PA or limited symptoms, and 24 (46%) of 52 participants denied any PA symptoms. All participants received current low-dose escitalopram or sertraline as the main PD regimen. The ratio of PA and non-PA was similar in the training set (35.1% vs 64.9%) and the test set (34.2% vs 65.8%).

### Panic Attack Prediction Model

**Figure 3:**
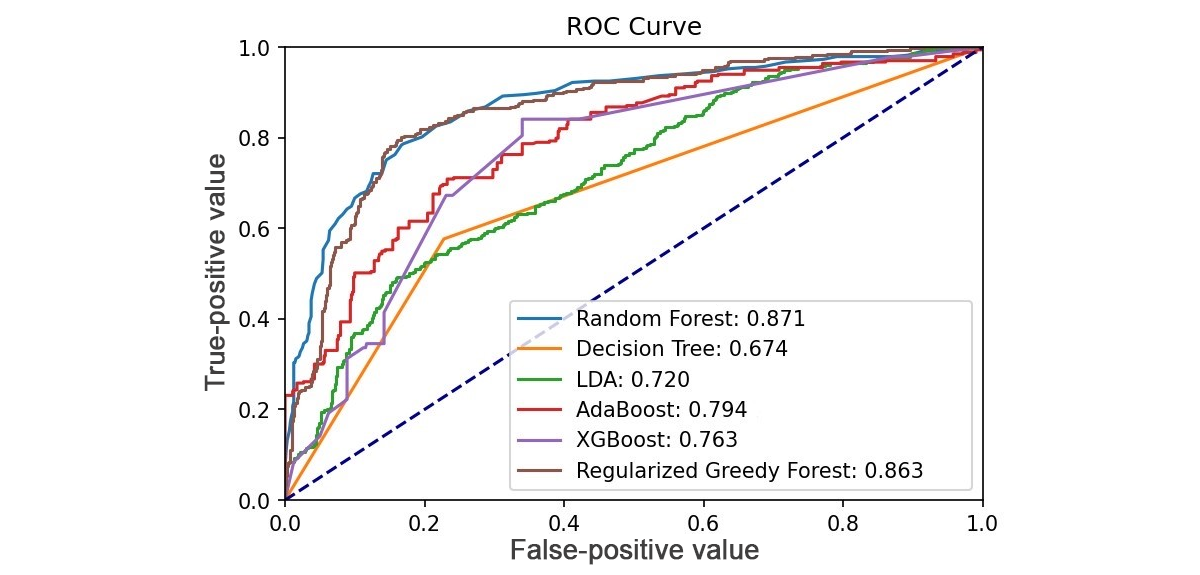
ROC curve analysis of prediction algorithms of test set. LDA: linear discriminant analysis; ROC: receiver operating characteristic.

We initially used data from the training phase to evaluate model performance, and the accuracy and F1 score of the implemented training set were as follows: random forest (0.975 and 0.968, respectively), decision tree (0.949 and 0.936, respectively), LDA (0.746 and 0.647, respectively), AdaBoost (0.838 and 0.792, respectively), XGBoost (0.702 and 0.458, respectively), RGF (0.945 and 0.928, respectively). Table 3 presents the test set performance. The random forest offered the highest accuracy compared to other models, whether in training or in testing models. The area under the receiver operating characteristic (AUROC) curve of each prediction algorithm is shown in Figure 3.

We also tested the model with different combinations of data sets, as shown in Table 4. These results show that the prediction performance of the all-feature model is better than that of the physiological-environment model or the questionnaire model alone.

**Table 3:** Test set performance of each model with all features.

Model	Accuracy	AUROC^a^	Specificity	Sensitivity	Precision	F1 score
Random forest	0.813	0.871	0.938	0.574	0.827	0.677
Decision tree	0.705	0.674	0.772	0.577	0.568	0.572
LDA^b^	0.722	0.720	0.850	0.474	0.622	0.538
AdaBoost^c^	0.746	0.794	0.872	0.505	0.672	0.576
XGBoost^d^	0.674	0.763	0.913	0.213	0.559	0.309
RGF^e^	0.800	0.863	0.920	0.568	0.788	0.660

^a^AUROC: area under the receiver operating characteristic.

^b^LDA: linear discriminant analysis.

^c^AdaBoost: adaptive boosting.

^d^XGBoost: extreme gradient boosting.

^e^RGF: regularized greedy forest.

**Table 4:** Test set performance of each model with various data set combinations.

Feature	Model	Accuracy	AUROC^a^	Specificity	Sensitivity	Precision	F1 score
All features	Random forest	0.813	0.872	0.938	0.574	0.827	0.677
Lifestyle and environment	RGF^b^	0.674	0.687	0.773	0.477	0.513	0.495
Questionnaire	RGF	0.771	0.843	0.858	0.617	0.712	0.661

^a^AUROC: area under the receiver operating characteristic.

^b^RGF: regularized greedy forest.

### Feature Importance

**Figure 4:**
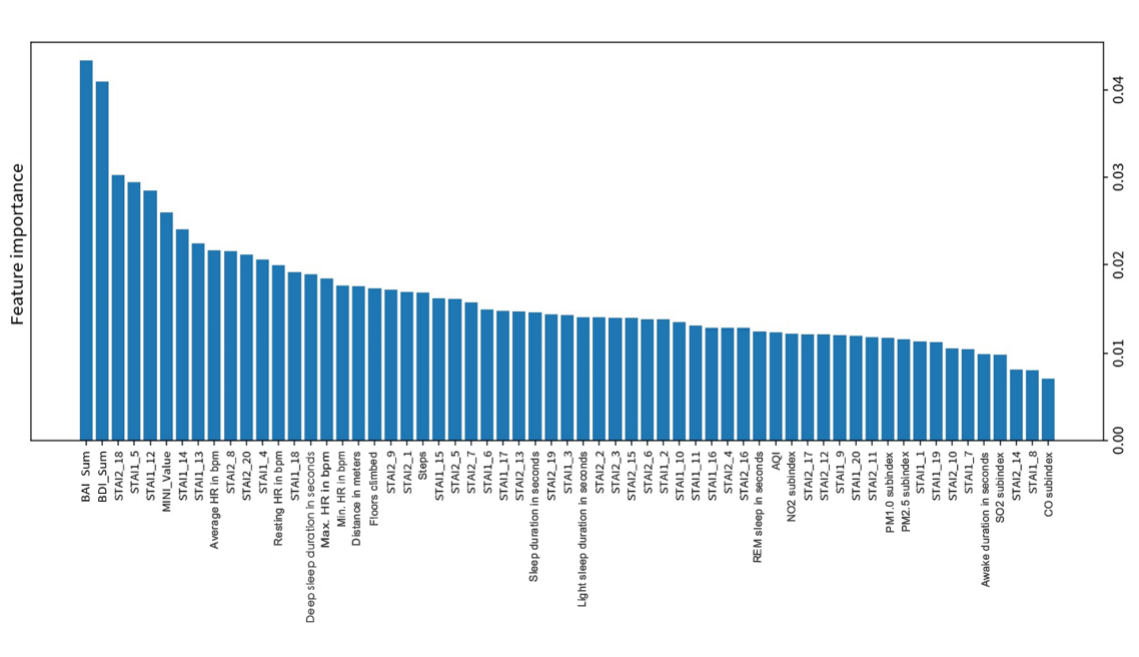
Feature importance of the all-feature model from a random forest. AQI: air quality index; BAI: Beck Anxiety Inventory; BDI: Beck Depression Inventory; bpm: beats per minute; MINI: Mini International Neuropsychiatric Interview; PM_1.0_: particulate matter 1.0 microns; PM_2.5_: particulate matter 2.5 microns; REM: rapid eye movement; STAI: State-Trait Anxiety Inventory.

Feature importance refers to a feature’s importance level in model prediction: the larger the number, the more critical the feature. Figure 4 shows the feature importance of the all-feature model. Questionnaire and physiological features, such as the BAI, BDI, STAI, MINI, average HR, resting HR, and deep sleep duration, were more critical than others in this prediction model.

## Discussion

### Principal Findings

PAs can be predicted 1 week before occurrence by machine learning through clinical questionnaires, physiological data, and environmental data. Random forests yielded the best prediction accuracy (81.3%) on the test set. Overall, the test set accuracy was 67.4%-81.3% for various machine learning algorithms. The feature importance ranking from high to low was clinical questionnaires, physiological data, and environmental data in the training set. The essential features for PA prediction were the BDI, BAI, STAI, MINI, HR in different states, and deep sleep duration. The prediction performance of the all-feature model was better than that of the physiological-environment model or the questionnaire model alone. This also highlighted that wearable devices detecting HR or deep sleep duration could be a potential tool to predict PAs.

### Study Strengths

To the best of our knowledge, this is the first PA prediction model study evaluated in *real life* with a full year of *continuous monitoring*. We also provided *multifactor features* for PA prediction, including physiological factors via smartwatches, clinical questionnaires, and environmental factors. We collected the questionnaire data via an *internet-based* mobile app, which is more accessible for most participants. Most participants gave positive feedback after learning to self-monitor their emotional and physiological states through wearables and regular questionnaires under supervision.

### Study Limitations

First, the sample size (N=59) was limited because this study required participants’ intensive cooperation. However, at the time of this study, 59 was a relatively large number in the known literature on using wearables for PA prediction [23,25]. Second, the prediction model was derived primarily from participants under regular medication in a single hospital. The performance would benefit from within-patient correlation; however, more external testing is needed for those patients unseen by the model. Third, the PA ground-truth labels were from the PDSS-SR questionnaires [32]. The participants’ memory recall could be biased while tracing back to previous events; labeling validity also depends on the participants’ understanding of the nature of PAs. To minimize these problems, we provided comprehensive psychoeducation to participants before this trial. Research teams used telephone follow-ups every 2 weeks to determine whether there were obvious outliers or missing data due to technical problems with the participants. Finally, according to the current study design, the PA prediction result applies only to patients with an established diagnosis of PD.

### Comparison With Prior Work

This study used multifactorial variables. Compared to previous PA or PD studies [13,33], our study combined questionnaire data with physiological and environmental data, resulting in superior prediction results as compared to a single data source (see Table 4). Prior work [21] focused on PA prediction was in clinician-monitored environments. However, wearables, such as smartwatches, and mobile apps [34,35] can be used in real-life situations, increasing ecological validity. In previous studies, the wearables’ testing duration was often days to weeks or cross-sectional [23], detecting few real-time PA events. Our study continued for 1 year and detected PA events in 1140 (35.09%) of all 3249 data points, a more balanced data distribution, making machine learning a possible tool for prediction.

In our experience, regular online questionnaires require intensive cooperation from participants and supervision by clinicians, which may be burdensome [36]. Wearable devices, however, are easier for autorecording with a real-time notification function. The use of combined methods for PA prediction needs further feasibility studies in actual clinical settings. Several studies have correlated the HRV to trait anxiety and depressiveness [37,38]. Thus, it is possible to merge the measurement of trait anxiety (STAI-T) and depressiveness (BDI) from questionnaires into wearables with an HRV-detecting function to provide information for prediction.

Our team also found that the AQI is less critical than questionnaires and wearable sensor data, which differs from the result, showing a significant relationship between air pollution and PAs in emergency visits [6]. The difference needs further evaluation because the nearest environmental monitoring station to the residential address may not reflect the actual location where each participant stayed. Using the Global Positioning System or air quality sensors located at individual participants’ homes is one way to address this problem.

### Clinical Suggestions

To better predict PAs, it is possible to use multifactorial items from clinical questionnaires and physiological and environmental data. Among these, clinical questionnaires are more crucial than their physiological-environmental counterparts. It is also beneficial to collect information from baseline anxiety and depression, trait anxiety, the number of comorbid psychiatric diagnoses, the average and resting HR, and deep sleep duration as a reference to predict recurrent PAs for patients with PD.

### Future Work

First, we will collect more participants to increase the effect size and sample heterogeneity. Currently, we do not clearly understand the correlation between PA symptoms and individual features. We suggest using an explainable model and combining questionnaires with real-time HRV data to establish a model to predict PAs hours before their occurrence.

### Conclusion

This prospective study introduced a 7-day prediction model for PAs based on machine learning using wearable devices, online questionnaires, and environmental data for a combinational assessment of PD, continuously monitoring samples from real-life settings for 1 year.

It is possible to predict PAs 7 days before the fact by using a combination of all data from questionnaires, physiological data, and environmental data. The prediction accuracy was 67.4%-81.3% for the test set from various machine learning algorithms, among which random forests offered the highest accuracy compared to other models. The prediction performance of the all-feature model is better than the physiological-environment model or questionnaire model alone. The features that contributed most to the prediction models are the BAI, BDI, STAI, MINI, average HR, resting HR, and deep sleep duration. However, current findings apply only to patients with an established diagnosis of PD. More external testing is also needed.

## Abbreviations

AdaBoost: adaptive boosting
AQI: air quality index
AUROC: area under the receiver operating characteristic
BAI: Beck Anxiety Inventory
BDI: Beck Depression Inventory
bpm: beats per minute
DI: diagnostic interview
DSM-5: *Diagnostic and Statistical Manual of Mental Disorders, 5th edition*
ECG: electrocardiogram
EEG: electroencephalogram
EMR: electronic medical record
fMRI: functional magnetic resonance imaging
HAM-A: Hamilton Anxiety Rating Scale
HAM-D: Hamilton Depression Rating Scale
HR: heart rate
HRV: heart rate variability
LDA: linear discriminant analysis
MINI: Mini International Neuropsychiatric Interview
PA: panic attack
PD: panic disorder
PDSS-SR: Panic Disorder Severity Scale (PDSS), Self-Report
PM_1.0_: particulate matter 1.0 microns
PM_2.5_: particulate matter 2.5 microns
PTSD: posttraumatic stress disorder
RGF: regularized greedy forest
REM: rapid eye movement
ROC: receiver operating characteristic
STAI-S: State-Trait Anxiety Inventory state anxiety
STAI-T: State-Trait Anxiety Inventory trait anxiety
XGBoost: extreme gradient boosting
